# Soybean Yield and Seed Composition Changes in Response to Increasing Atmospheric CO_2_ Concentration in Short-Season Canada

**DOI:** 10.3390/plants8080250

**Published:** 2019-07-27

**Authors:** Elroy R. Cober, Malcolm J. Morrison

**Affiliations:** Agriculture and Agri-Food Canada, Ottawa Research and Development Centre, Ottawa, ON K1A 0C6, Canada

**Keywords:** soybean, *Glycine max* (L.) Merr., breeding, climate change

## Abstract

From 1993, we have conducted trials with the same set of old to newer soybean cultivars to determine the impact of plant breeding on seed yield, physiological and agronomic characteristics, and seed composition. Since 1993, global atmospheric [CO_2_] increased by 47 ppm. The objective of our current analysis with this data set was to determine if there were changes in soybean seed yield, quality or phenology attributable to elevated atmospheric CO_2_ concentration (eCO_2_), temperature or precipitation. Additionally, we estimated genetic gain annually. Over 23 years, there was a significant increase in atmospheric [CO_2_] but not in-season average maximum or minimum temperatures, or average in-season precipitation. Seed yield was increased significantly by eCO_2_, higher precipitation and higher minimum temperatures during flowering and podding. Yield decreased with higher minimum temperatures during vegetative growth and seed filling. Seed oil and also seed protein plus oil concentrations were both reduced with eCO_2_. Phenology has also changed, with soybean cultivars spending less time in vegetative growth, while time to maturity remained constant. Over the 23 years of the study, genetic improvement rates decreased as [CO_2_] increased. Newer cultivars are not better adapted to eCO_2_ and soybean breeders may need to intentionally select for favourable responses to eCO_2_ in the future.

## 1. Introduction

Atmospheric carbon dioxide concentration has increased from 270 ppm, at the beginning of the industrial revolution (~1830), to 404 ppm at the present day (2016) [1,2]. Nearly 66% of total CO_2_ accumulation has occurred since the 1950s [3]. The International Panel on Climate Change (IPCC) estimated that even with serious carbon emission reductions, [CO_2_] will reach 480 ppm by 2050 [4]. In the atmosphere, CO_2_ absorbs long-wave radiated energy resulting in increased air temperature (T). This is referred to as the Greenhouse Effect. Since the beginning of the industrial revolution, global temperatures have risen by 0.9 °C and are predicted to continue to rise ~2.0 °C by 2050 [4].

In plants that only fix CO_2_ into 3-phosphoglycerate (C3 plants), increased [CO_2_] accelerates the rate of CO_2_ fixation in the chloroplast by enhancing the carboxylation reaction, while simultaneously inhibiting the oxygenation reaction of the enzyme ribulose-1, 5-biphosphate carboxylation/oxygenation (RuBisCO) [5]. The oxygenation reaction of RuBisCO is photorespiration. In theory, at 25 °C, a rise in [CO_2_] from 380 to 550 ppm would increase photosynthesis by 38% [6]. 

Fischer et al. [7] reported that an increase in [CO_2_] will be accompanied by an increase in T, which in many crops or locations will offset the positive aspects of greater photosynthetic responses toward higher yields. They estimated that a 100 ppm rise in [CO_2_] will result in a 10% rise in C3 plant crop yield globally, or 0.1% per ppm CO_2_. Hatfield et al. [8] determined that the predicted rise in T and [CO_2_] by 2050 will be detrimental to soybean yield in the southern US, while beneficial to yield in western and northern areas of production where the predicted rise in T will not exceed cardinal temperatures for growth and yield. Kimball [9] predicted a 21% increase in soybean seed yield from an increase in [CO_2_] from 380 to 550 ppm, or 0.12% per ppm CO_2_. From a meta-analysis of 111 controlled environment experiments, Ainsworth et al. [10] found that when CO_2_ was augmented from ambient to an average of 689 ppm, soybean leaf photosynthesis, plant biomass, and seed yield were 39, 37, and a 24% greater, respectively. Assuming an average initial ambient concentration of 380 ppm CO_2_, this resulted in a seed yield increase of 0.08% per ppm CO_2_. Sakurai, et al. [11] modeled US soybean production and estimated that yield increased by 110 kg ha^−1^ over 25 years (1980 to 2004) due to an increase in atmospheric [CO_2_] of 4.33%; or approximately 2.2 kg ha^−1^ ppm^−1^ CO_2_. 

To overcome the limitations of using growth chambers and greenhouses to study the influence of elevated atmospheric [CO_2_] (eCO_2_), the free-air concentration enrichment (FACE) facility was developed for in-field experiments. It consists of a circle of emitters that release CO_2_ on the upwind side of the circle, while sensors in the middle of the circle control the amount and direction of CO_2_ release with a microprocessor. Modern FACE systems can maintain eCO_2_ within ± 10% of the target concentration and large field plots with standard agronomic treatments can be used [12]. Morgan, et al. [13] reported that eCO_2_ from 373 to 550 ppm in SOYFACE resulted in a 2.5 kg ha^−1^ ppm^−1^ eCO_2_ increase in yield. Bunce [14] grew two soybean cultivars with +180 ppm over ambient CO_2_ in a FACE facility with daytime or 24 h eCO_2_ and found a mean 9.8 kg ha^−1^ ppm^−1^ and a 13.3 kg ha^−1^ ppm^−1^ increase in yield, respectively. 

Many plant breeders believe that selecting for yield in a slowly changing environment will produce cultivars capable of optimizing yield in those changed conditions. While eCO_2_ studies with different cultivars of soybean have found that total biomass increased as a result of increased photosynthesis, they did not show that seed yield increases at the same rate [15]. In a greenhouse experiment, Ziska et al. [16] found significant differences in yield response between soybean cultivars in response to an eCO_2_ from 390 to 700 ppm. Ainsworth et al. [10] stated that modern soybean cultivars may not be capable of capitalizing on eCO_2_, and that a wider range of soybean germplasm needs to be evaluated. Facility space and cost constraints limit the number of genotypes that can be compared under augmented eCO_2_ trials. Soybean breeders will need to be convinced of the necessity of selecting under eCO_2_ due to the increased costs that are incurred. 

Climate change as a result of eCO_2_ is associated with higher T and variations in precipitation patterns. From studies of long-term climate effects on soybean yield in north east China, Zheng et al. [17] concluded that climate changes have resulted in higher in-season daily maximum and minimum temperatures (Tmax and Tmin, respectively) and that yield responded positively to higher Tmin and negatively to higher Tmax. High cellular [CO_2_] results in stomata closure and improved water use efficiency [18]. In studies of water use in soybean, Purcell, et al. [19] reported an exponential-rise-to-a-maximum yield response to transpiration with 90% of asymptotic yield at 444 mm of water. Specht et al. [20] reported that in a dry year with irrigation set to replace evapotranspiration, soybean yield increased by 2.8 kg ha^−1^ mm^−1^ water. 

There is concern that eCO_2_ may result in dilution of essential nutrients in crop seeds due to a disproportionate increase in carbohydrates. A meta-analysis of multiple crop species found that there were lower concentrations of zinc and iron when crops were grown under eCO_2_ and in C_3_ non-legume crops, seed protein concentrations were also reduced [21]. Multi-year, multi-location FACE studies also predict a lowering of protein, micronutrients and vitamins in rice under eCO_2_ [22].

We conducted trials with a historical series of soybean cultivars to determine the influence of year of cultivar release on changes in yield (genetic gain), physiological and agronomic characteristics, the influence of seeding population on yield, the temporal nature of drought stress, and the effects of weed stress on genetic gain [23,24,25,26,27]. We have grown the same historical set of short-season soybean cultivars in replicated trials yearly from 1993 to 2016. Over that period atmospheric [CO_2_] has increased from 357 to 404 ppm [2], or 47 ppm. The objective of our research was to determine if soybean seed yield, composition and phenology has changed over time, and if so was it attributable to eCO_2_ and/or associated climate changes. Additionally, we also examined genetic gain (from the old to new cultivars) differences in response to annual changes in CO_2_ to test the hypothesis that changes in plant breeding techniques are required to directly select lines that can take greater advantage of predicted eCO_2_ versus simply ongoing yield testing in a changing environment. 

## 2. Results

**Figure 1:**
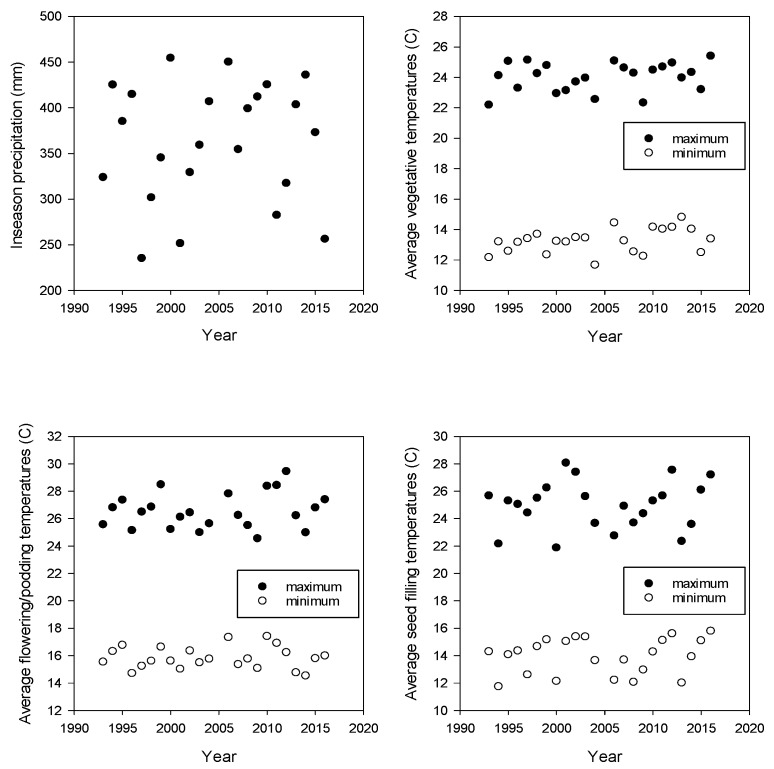
In season precipitation (mm), and average maximum and minimum in-season temperatures for the vegetative phase, the flowering to podding phase, and the seed filling phase; from 1993 to 2016.

Over the duration of the experiment from 1993 to 2016, atmospheric [CO_2_] increased significantly from 357 to 404 ppm or 2.0 ppm yr^−1^ (Table 1). In-season cumulative precipitation (pptn) ranged from 235 to 454 mm per year (Figure 1). Mean Tmax ranged from 21.9 to 29.5 °C over all three phenological phases (vegetative growth, flowering and podding, and seed filling), and mean Tmin temperatures ranged from 11.7 to 17.4 °C (Figure 1). 

The soybean life cycle was divided into three phases V, FP and SF and the number of days in each period determined each year on average over all cultivars since there was little difference among the cultivars. Changes in the duration of phenological stages were observed over the 23 years of the trial (Table 1). The time to first flower decreased over time throughout the duration of the trial (−0.35 d yr^−1^, *p* < 0.0001) while the total life cycle duration did not change. The proportion of the life cycle spent in vegetative growth decreased annually (−0.003 d yr^−1^, *p* < 0.0001).

In the investigation of weather parameters for collinearity, Tmax and Tmin were highly correlated during all three phases of development (Table 2). Tmax values were also correlated with precipitation in some phases of growth. Since long term analysis showed changes in minimum Ottawa summer temperatures, we only included Tmin parameters in the multiple linear regression analysis. Collinearity can also be detected by a parameter’s variance inflation factor and none of these were of concern (<5) (Table 3).

Seed yield was increased by some climate parameters and by plant breeding in our evaluation of old to newer cultivars grown over the 23 years of the experiment (Table 3). Genetic gain from plant breeding was estimated at 8.0 kg ha^−1^ yr^−1^ from the 59 years of breeding effort (1934 to 1992) that this set of cultivars represented. The increase in seed yield due to eCO_2_ was estimated at 4.3 kg ha^−1^ ppm^−1^ CO_2_. Seed yield increased with increasing pptn during all three phases: an estimated 2.2, 3.0 and 3.0 kg ha^−1^ mm^−1^ during vegetative (V), flowering and podding (FP) and seed filling (SF), respectively. Seed yield response to temperatures was not consistent. Seed yield increased with mean FP Tmin (251 kg ha^−1^ °C^−1^). Seed yield was reduced when mean Tmin increased during V (−158 kg ha^−1^ °C^−1^) and SF (−174 kg ha^−1^ °C^−1^) periods.

Plant breeding, over the 59 years that these 14 cultivars represented, resulted in decreased seed protein concentration (−0.58 g kg^−1^ yr^−1^) and greater seed oil concentration (0.45 g kg^−1^ yr^−1^) (Table 3). Seed oil concentration decreased with eCO_2_, (−0.25 g kg^−1^ ppm^−1^). Seed protein concentration increased with greater pptn (0.08, 0.17, 0.03 g kg^−1^ mm^−1^) during V, SF and FP, respectively. Seed protein was reduced by higher Tmin during V (−3.2 g kg^−1^ °C^−1^) and increased by higher Tmin during SF (3.4 g kg^−1^ °C^−1^). Seed oil was increased by increasing pptn during V (0.18 g kg^−1^ mm^−1^) and by increased Tmin during reproductive growth (FP 4.2 g kg^−1^ °C^−1^, SF 9.2 g kg^−1^ °C^−1^). The combination of seed protein plus oil concentration was reduced by eCO_2_ (−0.27 g kg^−1^ ppm^−1^). Seed protein plus oil was increased by increasing pptn during V and FP (0.24 g kg^−1^ mm^−1^, 0.16 g kg^−1^ mm^−1^, respectively), and by higher reproductive Tmin (FP 5.5 g kg^−1^ °C^−1^, SF 11.8 g kg^−1^ °C^−1^). Seed protein plus oil was decreased by higher Tmin during V (−4.7 g kg^−1^ °C^−1^, Table 3).

**Figure 2:**
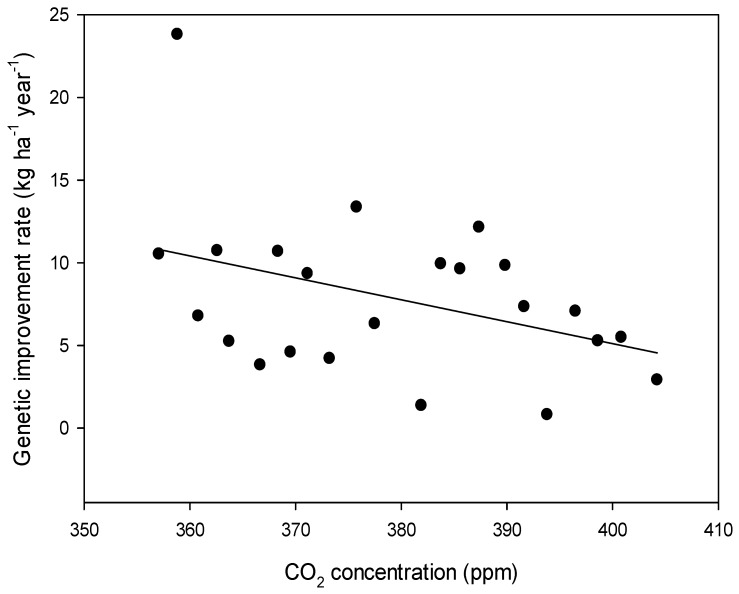
Rates of soybean genetic improvement, from 14 old to newer cultivars, measured over 23 years at Ottawa, Canada (1993 to 2016) versus annual atmospheric [CO_2_].

We calculated the genetic improvement rate for each year of the experiment for the 14 cultivars released from 1934 to 1992. The annual genetic improvement rates ranged from 0.8 to 23.8 kg ha^−1^ yr^−1^. When we examined the annual changes in genetic improvement rates using annual [CO_2_], we found that the rate of genetic improvement decreased as the [CO_2_] increased (Figure 2).

## 3. Discussion

Over the 23 years of this study, atmospheric [CO_2_ ]increased by an average of 2.0 ppm yr^−1^, using the Mauna Loa, CO_2_ measurements [2]. Our study estimated that soybean seed yield increased by 4.3 kg ha^−1^ ppm^−1^ CO_2_. Values in the literature for soybean yield increase due to eCO_2_ in free-air CO_2_ enrichment (FACE) experiments ranged from 2.2 [11] to 13.3 kg ha^−1^ ppm^−1^ [14]. Meta-analysis of controlled environment studies have predicted yield increases of 27 and 24% with a doubling in eCO_2_ [9,10]. It is worthy to note that our results have shown a significant increase in soybean yield under a relatively small eCO_2_ (a maximum of 47 ppm in the final year) while FACE or controlled environment studies have often been done with much higher increases in eCO_2_.

Over the 23 years of the experiment, annual in-season mean Tmax ranged from 23.3 to 27.3 °C and in-season Tmin ranged from 13.1 to 15.4 °C. While the mean maximum or minimum in-season temperature did not change statistically during our study, the Ontario Centre for Climate Impacts and Adaptation Resources [28] datasheet “Historical Climate Trends for Ottawa CDA, Ontario” reported a 2.0 °C increase in Average Summer Minimum T from 1890 to 2010 or 0.017 °C annually. Temperature during phenological periods affected soybean yield differently. Seed yield increased as Tmin increased (14.5 ranging to 17.4 °C) during FP (251 kg ha^−1^ degree^−1^). We have seen that lines selected for cold susceptibility during flowering yield less in the field [29], so perhaps there was some cold susceptibility in our set of cultivars which resulted in higher yields when FP temperatures were higher. Seed yield decreased as Tmin (11.7 ranging to 14.8 °C) increased during V (−158 kg ha^−1^ °C^−1^), and during SF (11.8 ranging to 15.8 °C) (−174 kg ha^−1^ °C^−1^). Soybean can be susceptible to heat stress generally in high stress situations [30] but our results suggest that moderate heat stress may also reduce seed yield. 

During the 23 years of this study, in-season pptn ranged from 235 to 454 mm but there was no significant trend evident over time. There was also no trend highlighted in the long term average in-season pptn (Average Summer Precipitation from 1890 to 2010, [28]. In our analyses across three growth phases, seed yield increased with increasing pptn (2.2 to 3.0 kg ha^−1^ mm^−1^, Table 3). Our previous work with this set of germplasm reported seed yield increased with pptn received during the FP and SF phase by 13.9 kg ha^−1^ mm^−1^ [25] although we did not separate the effects of eCO_2_ and T from pptn in that analysis. An estimate of soybean yield response to in-season precipitation in Wisconsin (5.0 kg ha^−1^ mm^−1^) [31] was closer to our current analysis. Specht et al. [20] irrigated to replace evapotranspiration and reported a soybean yield response of 2.8 kg ha^−1^ mm^−1^. Analysis of US soybean seed yield response to temperature and precipitation from 1994 to 2013, indicated regional responses but concluded that temperature changes were more important than precipitation changes [32]. When considering the partial R^2^ values for individual temperature and precipitation parameter effects on seed yield in our data set, the difference in importance of temperature and precipitation depended on growth phase but temperature was generally more important than precipitation (Table 3). 

The IPCC [4] estimates that by 2050 atmospheric [CO_2_] will increase to at least 480 ppm and global temperatures will rise by 2 °C. Hatfield et al. [8] pointed out that the effects of eCO_2_ and resulting warmer temperatures on soybean yield depends largely on latitude. The southern United States, with higher temperatures during critical phases of development will suffer to a greater extent by climate change than mid-west United States and Canadian production regions, where an increase in T will not exceed the critical maximum or cardinal T.

Over the 23 years of this study, the mean genetic improvement rate was 8.0 kg ha^−1^ year^−1^ which is somewhat lower than our previous estimates (11 to 16.6 kg ha^−1^ year^−1^) with short-season soybean [27,33]. When we examined annual estimates of genetic improvement compared to annual [CO_2_], genetic improvement rates dropped as [CO_2_] increased (Figure 2) indicating that newer cultivars are not better adapted to eCO_2_. Field crop studies estimating genetic improvement rates with a range of old to modern cultivars in a range of [CO_2_] are limited. A barley study with two cultivars representing 54 years of breeding found the new cultivar yielded more than the older cultivar under eCO_2_ [34]. Barley studies with up to four cultivars representing 93 years of breeding found that old cultivars were more responsive to eCO_2_ for grain yield [35,36]. A study with eight oat cultivars oat representing about 80 years of breeding reported that response to eCO_2_ was not related to year of release [37]. There is no consensus arising regarding genetic improvement rate response to eCO_2_ within cereals. In a soybean study to identify traits which were responsive to eCO_2_, Kumagai et al. [38] used a low planting density to conclude that, pod number per plant, and above ground biomass were as promising traits but the study did not use a set of old to new cultivars. In our genetic improvement rate studies with planting density, we found that low planting densities resulted in lower rates of genetic gain compared to high planting densities [26]. Taken together, good eCO_2_ performance under low planting density and old cultivar performing relatively better under low planting density, these studies suggest that old cultivars would respond best to eCO_2_, similar to the results of our current study under rising [CO_2_]. The same conclusion was reached by Ainsworth et al. [10] working with soybean. In order to benefit from eCO_2_, soybean breeders may need to deliberately select parents and progeny under eCO_2_. 

In 1934, when the first cultivar in our study was released the atmospheric [CO_2_] was 309 ppm. When we began our test, the oldest cultivar was already being grown in a CO_2_ environment 52 ppm greater than it was bred for and when the study ended CO_2_ was 95 ppm greater than at its release. Whereas the newest cultivar in our trial, released in 1992, was responding to a 49 ppm increase in CO_2_ by the end of the experiment. The actual change in eCO_2_ was much greater for the older cultivars than for the more recent ones which may have resulted in their greater yield improvement and a decrease in estimated genetic gain over the duration of the experiment. 

We found that seed composition has changed as a result of plant breeding where seed protein was reduced 0.58 g kg^−1^ yr^−1^ and seed oil increased 0.45 g kg^−1^ yr^−1^ (Table 3). Our previous work also found similar changes in seed composition due to plant breeding where seed protein was reduced 0.54 g kg^−1^ yr^−1^ while oil increased 0.45 g kg^−1^ yr^−1^ [23]. In addition to breeding effects, we found that eCO_2_ reduced seed oil (−0.25 g kg^−1^ ppm^−1^) and seed protein plus oil (−0.27 g kg^−1^ ppm^−1^) (Table 3). Meta-analyses have been carried out to examine eCO_2_ effects on seed composition. In one case, eCO_2_ did not affect soybean seed protein concentration [21] while another found a small reduction in seed protein [39]. Since seed protein and oil are the primary commercial products of oilseed soybean, a reduction in either or both components makes the soybean grain less valuable. In-season pptn and Ts also affected seed composition (Table 3). Seed protein was decreased by higher V pptn and higher Tmin during SF. Seed protein was increased by higher pptn during V, FP and SF phases, and by higher Tmin during SF. Seed oil was increased by higher pptn during V, and higher Tmin during SF and SP phases. In a study to estimate the effects of T and pptn on soybean protein and oil, increased Tmax during reproduction reduced protein and increased Tmin during reproduction was associated with increased oil [40]. When considering the combination of protein and oil, both [CO_2_] and minimum T during SF were important with eCO_2_ reducing, and high T increasing seed protein plus oil (Table 3). 

**Figure 3:**
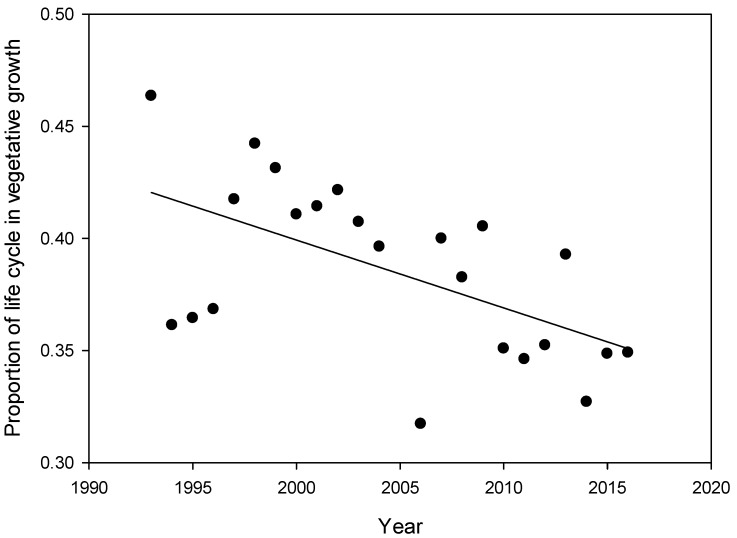
The proportion of time spent in vegetative growth for soybean cultivars grown from 1993 to 2016 at Ottawa, Canada.

Over the years of this study, we found that soybean genotypes flowered earlier but matured at the same time reducing the proportion of total time spent in vegetative growth (Table 1, Figure 3). In our study, eCO_2_ was the most important factor reducing the duration of the V portion of growth, accounting for almost one-third of the variation (Table 3). A review of soybean flowering time response to eCO_2_ reported two studies with delayed flowering, two with no change and one with accelerated flowering [41]. A FACE study reported delayed maturity under eCO_2_ with flowering significantly delayed in only one of three years [42]. A consensus has not developed on the role of eCO_2_ in phenology. Temperature has also been shown to affect flowering time [43]. Hatfield et al. [8] found that higher temperatures during the vegetative stage promoted growth but shortened the duration of soybean grown in the United States. Changes in phenology which increase the amount of time spent in reproductive growth have been shown to increase soybean yield [44,45]. Soybean breeding for yield improvement in the northern US has also produced this favourable change in phenology, where newer cultivars have a shorter V period [46]. The decrease in V duration over time whether caused by climate or indirectly through plant breeding, or both, may be important to account for in future climate change models predicting soybean growth and yield.

The IPCC has predicted that by 2050 global atmospheric [CO_2_] will reach 480 ppm and air T will rise by 2 °C. During the course of our study, [CO_2_] increased by 47 ppm. We have shown that eCO_2_ in Ottawa, Canada has resulted in higher soybean yield and lower seed oil and lower seed oil plus protein concentration. We found that over the duration of our study seed yield was also positively influenced by greater pptn, and higher Tmin during FP. These changes in climate are within the IPCC predictions. Phenology has also changed with eCO_2_ with soybean cultivars spending less time in V. 

One of the main advantages of our study was that it did not require costly equipment to elevate [CO_2_]. Over the 23 years of the study, genetic improvement rates fell with annual eCO_2_ indicating that photosynthetic mechanisms or plant architecture of older cultivars may have been more responsive to eCO_2_ than newer ones which would have been selected in a higher background atmospheric [CO_2_]. This is positive evidence that in order to capitalize on higher atmospheric [CO_2_] in the future, soybean breeders should be selecting parents and possibly also progeny that are more responsive to eCO_2_ despite the increased cost of this selection.

## 4. Materials and Methods 

Fourteen short-season cultivars were chosen to represent seven decades of soybean selection and cultivation from 1930 to 1992 (see [24] for cultivar details). These were grown in randomized complete block yield trials with four replications from 1993 to 2016 at Ottawa, ON, Canada (45°23′ N lat.). Plot row number varied with year, but there was a minimum of 4 rows spaced 40 cm apart and 6 m long in each plot. Seed of each cultivar was originally sourced from the plant breeding program at Ottawa and was renewed every 5 to 6 years. After the 2004 harvest, a seed processing error resulted in irrecoverable cultivar mixtures in the 2005 trial year. As a result, the data from 2005 was not included in this analysis and the cultivars were renewed for the 2006 trial year. 

Seed was inoculated with *Bradyrhizobium japonicum* and sown at a target population of 50 seeds m^−2^ to a depth of 1.5 to 2.0 cm using the same seeder for the duration of the experiment. The target seeding date was May 20th each year. *P* and K fertilizer was broadcast pre-plant and incorporated according to soil tests. Weeds were controlled using recommended herbicides and manual hoeing during the growing season. Until 2011, the experiment was grown on a Grenville loam (Cryochrepts, Eutrochrepts). Post 2011, the experiment was grown on a Matilda sandy loam (Cryochrepts, Eutrochrepts, Hapludolls). Mean phenology over all cultivars was used to break each growing season into the vegetative (V, planting to first flower), flowering to podding (FP, first flower to full pod), and seed filling (SF, full pod to full maturity) periods. The proportion of time spent in V growth was calculated as [V/(V + FP + SF)]. At maturity, plots were combine harvested, the seed was cleaned and weighed and the seed yield adjusted to 13% moisture by weight. Protein and oil concentration were measured each year using a near infrared reflectance (NIR) spectrophotometer (Infratec 1241 Grain Analyzer, FOSS Eden Prairie, MN) and reported on a dry matter basis.

In-season (May to September) daily temperature maximum and minimum, (Tmax, Tmin) and precipitation (pptn) were observed at a nearby weather station. Atmospheric CO_2_ concentration data were from the annual mean Mauna Loa [CO_2_] observations [Dr. Pieter Tans, NOAA/ESRL (www.esrl.noaa.gov/gmd/ccgg/trends/) and Dr. Ralph Keeling, Scripps Institution of Oceanography (scrippsco2.ucsd.edu/)]. Over the 23 years of the trial, annual changes in [CO_2_], in-season average Tmax and Tmin, and in-season pptn were determined from a linear relationship of the parameter over years (SAS, Cary NC). Linear regression parameters were considered significant when *P* < 0.05. Changes in mean phenology over the 23 years of the trial were examined by using linear regression (SAS, Cary NC) for the V period, the total time to maturity, as well as the proportion of time in V growth. The genetic improvement rate was determined through regression of seed yield on cultivar of year of release. Genetic improvement estimates were plotted versus annual [CO_2_] to visualize this relationship. 

Collinearity between parameters was examined before carrying out multiple linear regression using Pearson correlation analysis in Proc Corr of SAS. A moderate correlation was declared when 0.3 < |r| ≤ 0.7 and strong correlations when 0.7 < |r| ≤ 1.0. A threshold of |r| < 0.5 was used to exclude parameters from multiple linear regression following Wilmsmeyer et al. [47]. Stepwise multiple linear regression (Proc Reg, STEPWISE, SAS, Cary NC) was used to investigate the factors that were significant in determining soybean seed yield and composition including genetic improvement rate (as year of release), [CO_2_], and the climate parameters, pptn, average Tmax and average Tmin during the three phenological phases, V, FP, and SF. The stepwise multiple regression process adds significant (*p* < 0.15) parameters to the model and provides partial R^2^, mean effects, and standard errors for each significant parameter as they are added. Collinearity was examined using the variance inflation factor where values > 10, or more conservatively >5, indicate problems with collinearity [48].

## Figures and Tables

**Table 1:** Linear rate of change (slope) in climate parameters over 23 years (1993 to 2916), standard error of the estimate (SE) for the slope and significance of the linear regression (*p*-value) for [CO_2_] (ppm), in-season precipitation (pptn, mm), in-season average maximum and minimum daily temperature (Tmax, Tmin, respectively, °C) and proportion of time spent in vegetative growth (Vprop).

Climate Parameter	Linear Rate of Change	SE	*p*-Value
[CO_2_]	2.00	0.006	<0.0001
In-season PPTN	--	--	ns
In-season mean Tmax	--	--	ns
In-season mean Tmin	--	--	ns
Days to first flower	−0.35	0.027	<0.0001
Days to maturity	--	--	ns
Vprop	−0.0030	0.0002	<0.0001

-- Model parameters and standard error were not significant (ns, *p*-value) therefore were not included in the table.

**Table 2:** Pearson correlation coefficients between weather variables from 1993 to 2016 at Ottawa, Canada observed from soybean phenology stages. Correlations exceeding the limit for collinearity (| r | > 0.5) are shaded dark grey while moderate correlations are shaded light grey (0.3 < | r | < 0.5).

	[CO_2_]	V ppt	FP ppt	SF ppt	V Tmax	FP Tmax	SF Tmax	V Tmin	F PTmin
V ppt	−0.230								
FP ppt	−0.014	0.017							
SF ppt	0.190	−0.204	−0.066						
V Tmax	0.223	−0.583	−0.021	−0.050					
FP Tmax	0.189	−0.511	−0.262	0.665	0.665				
SF Tmax	0.090	−0.322	−0.489	0.111	0.111	0.366			
V Tmin	0.367	−0.058	−0.042	−0.090	0.525	0.364	−0.079		
FP Tmin	0.004	−0.198	−0.040	0.112	0.450	0.745	0.116	0.110	
SF Tmin	0.172	−0.349	−0.467	−0.080	0.106	0.341	0.906	−0.035	0.129

Phenology stages: V = vegetative, FP = flowering and podding, and SF = seed filling. Weather parameters: ppt = precipitation, Tmax = mean maximum temperature °C, Tmin = mean minimum temperature °C.

**Table 3:** Stepwise multiple linear regression analysis of genetic gain due to plant breeding (genetic gain, kg ha^−1^ yr^−1^)), [CO_2_] increase, and weather parameters affecting soybean seed yield, proportion of time spent in vegetative growth (Vprop), protein, oil and oil plus protein (g kg^−1^) from trials of 14 old to newer cultivars grown from 1993 to 2016 at Ottawa, Canada.

**Variable ^a^**	**Seed Yield (kg ha^−1^)**						**Vprop**					
	**Partial R2**	**Estimate**	**SE**	**T Ratio**	***p*-Value**	**Variance Inflation Factor**	**Partial R2**	**Estimate**	**SE**	**T Ratio**	***p*-Value**	**Variance Inflation Factor**
Intercept		−15146	3056	−4.96	<0.0001	0		1.278	0.044	28.78	<0.0001	0
Genetic gain	0.057	8.0	1.4	5.77	<0.0001	1.00						
[CO_2_], ppm	0.006	4.3	2.2	1.92	0.0556	1.43	0.315	−0.001	0.00001	−10.98	<0.0001	1.31
V pptn, mm	0.006	2.2	0.9	2.28	0.0233	2.54						
FP pptn, mm	0.011	3.0	0.9	3.32	0.0010	2.22	0.088	−0.0003	0.00003	−9.41	<0.0001	1.50
SF pptn, mm	0.040	3.0	0.6	4.82	<0.0001	1.47	0.118	−0.0003	0.00003	−11.89	<0.0001	1.16
V Tmin, °C	0.031	−158	37	−4.19	<0.0001	1.28	0.065	−0.014	0.002	−8.12	<0.0001	1.28
FP Tmin, °C	0.071	251	36	6.93	<0.0001	1.18	0.097	−0.013	0.002	−7.97	<0.0001	1.06
SF Tmin, °C	0.265	−174	36	−4.92	<0.0001	2.98	0.004	−0.002	0.001	−2.00	0.0459	1.57
Model *p*-value	<0.0001						<0.0001					
R2	0.49						0.69					
Adjusted R2	0.47						0.68					
RMSE	463						0.021					
**Variable**	**Seed Protein (g kg^−1^)**						**Seed Oil (g kg^−1^)**					
	**Partial R2**	**Estimate**	**SE**	**T Ratio**	***p*-Value**	**Variance Inflation Factor**	**Partial R2**	**Estimate**	**SE**	**T Ratio**	***p*-Value**	**Variance Inflation Factor**
Intercept		1500	95.2	15.79	<0.0001	0		−803	10.6	−7.57	<0.0001	0
Genetic gain	0.306	−0.58	0.05	−12.51	<0.0001	1.00	0.151	0.45	0.005	9.01	<0.0001	1.00
[CO_2_], ppm							0.029	−0.25	0.007	−3.40	0.0008	1.15
V pptn, mm	0.006	0.08	0.03	2.78	0.0058	2.13	0.106	0.18	0.003	6.67	<0.0001	1.54
FP pptn, mm	0.062	0.17	0.03	5.95	<0.0001	2.14						
SF pptn, mm	0.005	0.03	0.02	1.54	0.1242	1.46						
V Tmin, °C	0.024	−3.24	1.14	−2.85	0.0047	1.05						
FP Tmin, °C							0.021	4.21	0.126	3.34	0.0009	1.11
SF Tmin, °C	0.005	3.41	1.17	2.93	0.0037	2.91	0.131	9.15	0.084	10.93	<0.0001	1.28
Model *p*-value	<0.0001						<0.0001					
R2	0.41						0.44					
Adjusted R2	0.40						0.43					
RMSE	15.4						16.7					
**Variable**	**Seed Protein Plus Oil (g kg^−1^)**											
	**Partial R2**	**Estimate**	**SE**	**T Ratio**	***p*-Value**	**Variance Inflation Factor**						
Intercept		728	13.5	5.41	<0.0001	0						
Genetic gain	0.010	−0.13	0.006	−2.05	0.0417	1.00						
[CO_2_], ppm	0.095	−0.27	0.010	−2.77	0.0060	1.42						
V pptn, mm	0.039	0.24	0.004	6.35	<0.0001	2.00						
FP pptn, mm	0.035	0.16	0.004	4.37	<0.0001	1.83						
SF pptn, mm												
V Tmin, °C	0.019	−4.72	0.165	−2.87	0.0044	1.23						
FP Tmin, °C	0.020	5.49	0.161	3.41	0.0007	1.18						
SF Tmin, °C	0.093	11.76	0.138	8.50	<0.0001	2.28						
Model *p*-value	<0.0001											
R^2^	0.31											
Adjusted R^2^	0.29											
RMSE	20.6											

^a^ Variables were included in the model if the *p*-value < 0.15. Phenology stages: V = vegetative, FP = flowering and podding, and SF = seed filling. Weather parameters: pptn = precipitation (mm), Tmin = mean minimum temperature °C.
